# The Native Microbiome Member *Chryseobacterium* sp. CHNTR56 MYb120 Induces Trehalose Production via a Shift in Central Carbon Metabolism during Early Life in *C. elegans*

**DOI:** 10.3390/metabo13080953

**Published:** 2023-08-18

**Authors:** Tanisha Jean Shiri, Charles Viau, Xue Gu, Lei Xu, Yao Lu, Jianguo Xia

**Affiliations:** 1 Institute of Parasitology, McGill University, Sainte-Anne-de-Bellevue, QC H9X 3V9, Canada; tanisha.shiri@mail.mcgill.ca (T.J.S.); charles.viau@mail.mcgill.ca (C.V.); xue.gu@mcgill.ca (X.G.); lei.xu5@mcgill.ca (L.X.); 2 Department of Microbiology and Immunology, McGill University, Montreal, QC H3A 2B4, Canada; yao.lu5@mail.mcgill.ca

**Keywords:** aging, microbiome, metabolomics

## Abstract

Aging is the system-wide loss of homeostasis, eventually leading to death. There is growing evidence that the microbiome not only evolves with its aging host, but also directly affects aging via the modulation of metabolites involved in important cellular functions. The widely used model organism *C. elegans* exhibits high selectivity towards its native microbiome members which confer a range of differential phenotypes and possess varying functional capacities. The ability of one such native microbiome species, *Chryseobacterium* sp. CHNTR56 MYb120, to improve the lifespan of *C. elegans* and to promote the production of Vitamin B6 in the co-colonizing member *Comamonas* sp. 12022 MYb131 are some of its beneficial effects on the worm host. We hypothesize that studying its metabolic influence on the different life stages of the worm could provide further insights into mutualistic interactions. The present work applied LC-MS untargeted metabolomics and isotope labeling to study the impact of the native microbiome member *Chryseobacterium* sp. CHNTR56 MYb120 on the metabolism of *C. elegans*. In addition to the upregulation of biosynthesis and detoxification pathway intermediates, we found that *Chryseobacterium* sp. CHNTR56 MYb120 upregulates the glyoxylate shunt in mid-adult worms which is linked to the upregulation of trehalose, an important metabolite for desiccation tolerance in older worms.

## 1. Introduction

Biological aging is the progressive decline of cellular, physiological, and metabolic functions, leading to an increase in vulnerability to diseases or even death. Age-related decreases in metabolic activities affect nutrient utilization, waste removal, and energy production [1]. Loss of homeostasis during aging is due to the dysregulation of key signaling pathways such as mTOR, AMPK, and IGF-1, which work by sensing metabolic stress [2,3]. Certain hub metabolites, such as nicotinamide adenine dinucleotide, reduced nicotinamide dinucleotide phosphate, α-ketoglutarate, and β-hydroxybutyrate, have been identified at the intersections of these signaling pathways [4]. In addition to this, the gut microbiome is actively being established as a key modulator of aging [5]. Ongoing aging research aims to better-understand the underlying mechanisms of these age-related factors to identify potential drug targets or develop microbiome-based supplements to help delay or prevent age-related diseases. 

Although the correlation of the gut microbiome with aging is well-established in humans, obtaining mechanistic insights has been challenging due to the complexity of the human gut microbiome community [6]. Thus, model organisms have been widely used to study the aging phenotype based on their shared pathways and epigenetic cues with humans [2,3,7]. The discovery of the insulin-like receptor encoding DAF-2 in *Caenorhabditis elegans* (*C. elegans*) established it as a tractable model to study aging [8,9]. The insulin-like signaling system, which regulates whether the worm enters its reproductive cycle or arrests development at the dauer stage, was found to be analogous with the metabolic control of mammalian longevity via caloric restriction. Apart from this, its short lifespan of approximately 3 weeks allows researchers to track changes right from the larval stage to the late adult stages, making *C. elegans* a viable model for aging studies. The *C. elegans* model has played a key role in the discovery of several aging-related pathways [8,10]. 

The metabolic signatures of long-living worms have been repeatedly associated with dietary restrictions and the modulation of carbohydrate metabolism and amino acid catabolism [11]. Increased dietary glucose consumption has been linked with reduced lifespan [12]. After the cellular absorption of glucose, it is either funneled to the glycolytic pathway or stored as an energy source [13]. While mammals store energy in the form of glycogen, *C. elegans* can store energy in the form of both glycogen and trehalose [14]. It was demonstrated that when glycogen storage is reduced, a metabolic shift is observed which leads to an alternate energy storage route in trehalose [14]. Consequently, reduced glycolytic activity has been correlated with lifespan expansion, as seen in studies that used RNAi to knock down glucose phosphate isomerase 1, or *fgt-1* [13]. The causal link between lifespan reduction and increased glucose levels can be attributed to several mechanistic factors, such as mitochondrial dysfunction with prolonged exposure, which activates the mitochondrial unfolded-protein response [15], or a reduction in the oxidative phosphorylation pathway and reactive oxygen species signals [12,16]. The changes in carbohydrate metabolism have been further linked to the glyoxylate shunt and gluconeogenesis pathway [11] The glyoxylate shunt is an anaplerotic pathway that regenerates important TCA cycle intermediates under conditions of reduced glycolytic activity. Furthermore, due to its role in replenishing glucose levels downstream of gluconeogenesis, it was found to be directly involved in trehalose production [17,18]. Using isotope labeling metabolomics, a system-wide coordinated shift in glycolysis towards serine and purine metabolism was discovered in a Drosophila model of aging [7]. However, in *C. elegans*, a similar system-wide coordination of metabolic processes that directly affect aging and, more so, the changes in the production of specific metabolites throughout the lifecycle of the worm, has not been established. 

The native microbiome’s effect on longevity has been extensively studied, with some specific metabolites of interest identified. For instance, it has been shown that bacterial processing of metformin could directly alter the methionine metabolism in the host worm and, consequently, increase lifespan [19]. Similarly, it was also found that the activity of some cancer therapeutics were affected by the upregulation of certain bacterial ribonucleotide metabolism genes [20]. Recent years have seen increasing research efforts to characterize the composition of the native microbiome of *C. elegans* [21,22] as well as the functional capacities encoded within their genomes [23]. The influence of the native microbiome members on worm gene expression profiles has been studied across the developmental and adult stages [24,25]. It was demonstrated that a coordinated production of Vitamin B6 was facilitated in worms grown on a combination of *Comamonas* sp. 12022 MYb131 and *Chryseobacterium* sp. CHNTR56 MYb120 [26]. However, the influence of the native microbiome on metabolic processes that directly affect aging and, more so, on the production of specific metabolites throughout the lifecycle of the worm, has not been well-captured. Furthermore, most studies focused on amino acid and fatty acid profiles of worms, and the influence of the native microbiome on central metabolism is not clear. The present work aims to address this gap by performing a combination of untargeted or global metabolomics analysis and isotope labeling to identify the set of age-related metabolic signatures that are significantly influenced by the native microbiome. For this purpose, our study focused on the native microbiome strain *Chryseobacterium* sp. CHNTR56 MYb120.

## 2. Materials and Methods

### 2.1. Bacterial Growth Culture

*E. coli* OP50 and *Chryseobacterium* sp. CHNTR56 MYb120 colonies were grown overnight at 37 °C and 28 °C, respectively, in Luria–Bertani (LB) broth. In total, 50 µL of the overnight cultures was inoculated in 5 mL of M9 minimal salts. These were further grown for 16 h to their respective mid-log phase and seeded onto peptone-free NGM (NGMPF) plates with a targeted concentration of OD_595nm_ of 2.0 on the plates to normalize the bacterial growth on each plate.

### 2.2. Maintenance of C. elegans Worms

For our study, the *C. elegans* CF512 (rrf-3; fem-1) strain was used, which are sterile at 25 °C, making them better-suited for lifespan-based experiments [27]. CF512 worms maintained at 21 °C were synchronized to segregate eggs from adult worms [26]. Briefly, 16.6% alkaline bleach (*v*/*v*) was used to kill the adult worms in addition to sanitizing them. The eggs were isolated via two rounds of washing with M9 buffer, followed by centrifugation in 60% (*v*/*v*) sucrose in water gradient solution at 400× *g* for 6 min at room temperature. After washing with M9 buffer, the isolated eggs hatched overnight into L1 worms on a rocking machine. Around 2000 of these hatched L1 worms were then transferred to each NGMPF plate seeded with the respective bacterial group, as mentioned in Section 2.1, and the plates were incubated at 25 °C to prevent progeny production. 

### 2.3. Lifespan Assay

Worms were allowed to grow on *E. coli* OP50 or *Chryseobacterium* sp. CHNTR56 MYb120 seeded NGMPF plates and were transferred to fresh plates every two days. The number of surviving, dead, and censored worms were counted and tabulated at these transfer time points. The worms were gently prodded with a platinum wire to distinguish between live and dead worm responses to stimuli [28]. The worms that were dead or missing from the plate were censored from the analysis, as our focus was longevity [29]. The percentage of survival was calculated using OASIS2 (Online Application for Survival Analysis; https://sbi.postech.ac.kr/oasis2/introduction/ (accessed on 15 February 2023)) [30]. 

### 2.4. Worm Motility (Head Thrashing) Assay

To measure the difference in motility, 30 synchronized L1 worms were transferred to *E. coli* OP50 or *Chryseobacterium* sp. CHNTR56 Myb120 seeded NGMPF plates and were allowed to grow to the adult day 1 stage at 25 °C. At this stage, the worms were picked and deposited onto a drop of 50 µL ddH_2_O on a glass slide and were allowed to swim for 1 min to wash off any bacteria. Each worm was then individually picked and deposited onto 50 µL of M9 buffer on a slide and was allowed to swim for 30 s to become acclimatized. After this, the number of times the worms thrashed their heads back and forth was counted over 1 min. 

### 2.5. Worm Heat Resistance Assay

Forty-five L1 worms were grown in triplicate to adult day 1 stage on respective *E. coli* OP50 or *Chryseobacterium* sp. CHNTR56 Myb120 seeded NGMPF plates at 25 °C, as specified in the previous sections. These young-adult worm plates were shifted to a 37 °C incubator to induce heat stress. The plates were individually checked for dead worms every hour, which were tabulated and discarded. 

### 2.6. Global Metabolomics and Stable Isotope Labeling of C. elegans Worms

Worms were labeled indirectly via their bacterial diet [26,31]. Cultures of *E. coli* OP50 and *Chryseobacterium* sp. CHNTR56 Myb120 were first labeled using 0.2% uniformly labeled glucose (U-^13^C) supplemented with M9 minimal salt media and unlabeled control cultures were grown in parallel with 0.2% of unlabeled glucose (^12^C). Cultures were grown in triplicate for each experimental group up to their respective mid-log phase, including the labeled cultures, to account for pseudo-steady labeling. All the bacterial growth conditions were the same as those described in Section 2.1. At this point, the labeled and unlabeled cultures were spread on NGMPF plates to grow bacterial lawns with a targeted concentration of OD_595nm_ of 2.0 on the plates and were incubated at 37 °C for *E. coli* OP50 and 28 °C for *Chryseobacterium* sp. CHNTR56 Myb120 over a period of 16 h. Since labeling of the worms throughout their lifecycle was desired, 60 plates were prepared for labeled and unlabeled cultures of *E. coli* OP50 and *Chryseobacterium* sp. CHNTR56 Myb120, and for an experimental period of 12 days there were five transfer points every 2 days to maintain fresh food source for the worms (triplicates × 2 × 2 × 5 = 60 plates). As described in Section 2.2., 2000 synchronized L1 CF512 worms were added to the required number of starting plates and incubated at 25 °C. Worms were periodically transferred every two days and sampled at four sampling points for the L4-young adult stage, young adult day 1, middle-aged adult day 6, and older day 10 worms. Sampled worms were centrifuged, washed with M9 buffer twice, and the pellets were frozen pending extraction and downstream LC-MS analysis.

### 2.7. Metabolite Extraction

The next step was to extract metabolites from the worms. Due to their hard outer cuticle, the worms needed to first be broken open and homogenized. To perform this, the frozen worm pellets were mixed with 1.0 mL of cold 80% methanol (*v*/*v*) in water to first quench the metabolism of the worms, followed by mechanical shearing in a bead mill where worms added to ceramic bead tubes were homogenized for 30 s followed by incubation on ice for 1 min. This was repeated four times to thoroughly homogenize the samples. Samples were then centrifuged at 14,000× *g* for 10 min at 4 °C to separate the worm debris and to extract metabolites. The pellet was frozen for future protein concentration analysis to normalize the samples based on protein content using a bicinchoninic (BCA) assay. The supernatant was dried in a SpeedVac to further concentrate the metabolites and to evaporate the added methanol. The samples were then reconstituted in 150 µL 60:40 (*v*/*v*) acetonitrile/water and filtered using a Nanosep 0.22 µm centrifugal filter. A total of 40 µL of each sample was used to generate a pooled quality control (QC) sample to monitor system reproducibility. Samples were then transferred to MS vials for analysis.

### 2.8. LC-MS and MS/MS

The UHPLC-MS/MS measurement was performed using Ultimate 3000 liquid chromatography coupled with Q-Exactive Orbitrap HRMS (Thermo Fisher Scientific, Waltham, MA, USA) based on the previously described method [26]. A chromatographic separation was performed on a Thermo Hypersil GOLD C18 column (100 mm × 2.1 mm, 1.9 µm) maintained at 40 °C, with a flow rate of 0.4 mL/min. The samples were kept under 4 °C and injection volume was 5 µL. The gradient elution was composed of mobile phase A (0.1% formic acid in water) and mobile phase B (0.1% formic acid in acetonitrile) and performed for 22 min, as shown in Supplementary Table S1. The Q-Exactive Orbitrap HRMS was equipped with heated electrospray ionization (HESI) source using the following source parameters: sheath gas flow—55 arb (arbitrary units); aux gas flow—10 arb; spray voltage—+4.0/−3.5 KV; capillary temperature—350 °C; S-lens RF level—55; aux gas heater temperature—300 °C. For MS, resolution was set as 70,000 and Scan range was 70 to 1000. For MS/MS, data-dependent acquisition (DDA) mode was used, and the top 10 precursors were fragmented by 15, 30, and 45 collision energy values. 

### 2.9. Statistical Analysis

The raw LC-MS spectral files were centroided and converted to mzML format using Proteowizard [32,33]. The .mzML files were preprocessed using OptiLCMS and the auto-optimized parameters were used to generate a peak intensity table [34]. The peak intensity table was then subjected to data correction using MetaboAnalyst 5.0 [35]. About 10% of the features were filtered out based on the Interquartile Range (IQR) and any features whose relative standard deviation (RSD) was greater than 25% in the QC samples were also filtered out. Next, the samples were normalized based on weight of the protein, which was determined via the protein concentration assay. Principal component analysis (PCA) was used to examine patterns of separation. Next, PLS-DA was performed to identify the important metabolites associated with the separation of the different age groups. The metabolites that contributed most to the separation between age groups were retained based on the variable importance in projection (VIP) scores. A metabolite was considered to be a potential candidate for age-related modulation if its VIP score was above 2. The statistical significances were further tested using analysis of variance (ANOVA). Next, the biological significance of these metabolites was cross-checked against the DrugAge database entries for compounds associated with aging in *C. elegans* [36], and through manual literature survey.

For the isotope labeling data, the .mzML files preprocessed by OptiLCMS were analyzed using X13CMS [37]. The mass isotopologue distributions (MID) of the peaks were then plotted for all the life stages. Labeling extent (LE) for all metabolites of the central carbon metabolism was calculated as described in [7]. Briefly, LE is the level of enrichment of the added ^13^C label in each metabolite. This is the ratio of the peak intensity of the monoisotopic peak to the sum total of the peak intensities of all the isotopologue peaks. 

The MS/MS data processing was accomplished using the MetaboAnalystR 4.0 software package (https://github.com/xia-lab/MetaboAnalystR (accessed on 10 March 2023)) [32,33,34] based on its latest online tutorial (https://www.metaboanalyst.ca/docs/RTutorial.xhtml (accessed on 10 March 2023)). Briefly, the .mzML DDA spectra data files were deconvoluted through a database-assisted method and subsequently annotated against the KEGG pathway library. The annotation results are included in Supplementary Table S3.

### 2.10. Functional Analysis

Functional analysis was performed using the web implementation of *mummichog* on MetaboAnalyst [35,38,39]. The respective peak intensity table for each life stage was uploaded to the “functional analysis” module on MetaboAnalyst. The data were first quantile normalized and log transformed. Next, *mummichog* version 2 was implemented for a *p*-value cut-off of 0.05 against the KEGG pathway library for *C. elegans*. The results are included in Supplementary Table S2.

## 3. Results

### 3.1. Chryseobacterium sp. CHNTR56 MYb120 Increases Lifespan and Heat-Stress Resistance but Reduces Motility Functions in C. elegans

**Figure 1:**
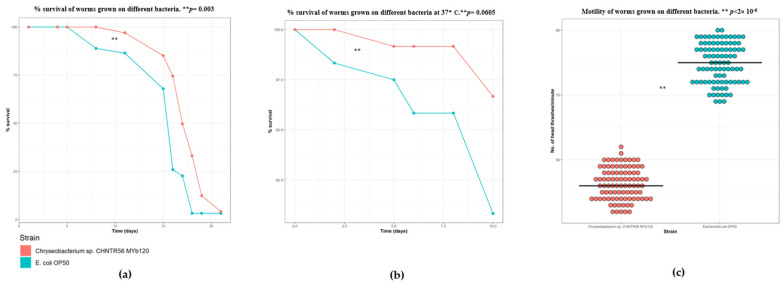
*Chryseobacterium* sp. CHNTR56 MYb120 positively influences worm lifespan and heat resistance, but not motility. (**a**) Lifespan analysis results showing lifespan extension of CF512 animals grown on *Chryseobacterium* sp. CHNTR56 MYb120 versus *E. coli* OP50; (**b**) heat resistance assay results showing increased fitness of animals grown on *Chryseobacterium* sp. CHNTR56 MYb120 versus *E. coli* OP50; (**c**) motility assay demonstrating lesser number of head thrashes per minute when animals were grown to the adult day 1 stage on *Chryseobacterium* sp. CHNTR56 MYb120 compared with *E. coli* OP50.

In this study, CF512 animals, which are sterile at 25 °C, were used instead of the widely used Fluorodeoxyuridine (FudR) method which helps maintain synchronized populations of *C. elegans* [40]. The median lifespan for CF512 worms is typically 20 to 22 days [27,41]. The results of the lifespan analysis assay revealed an 18% increase in percent survival (*p*-value = 0.0003, Mantel–Cox test) of CF512 worms grown on *Chryseobacterium* sp. CHNTR56 MYb120 as compared with those grown on *E. coli* OP50 (Figure 1a). They are comparable to a previous study with N2 worms grown on *Chryseobacterium* sp. CHNTR56 Myb120, which showed a 20% increase in lifespan [25]. When studying the difference in heat-stress resistance of young *C. elegans*, adult day 1 worms grown on *Chryseobacterium* sp. CHNTR56 MYb120 and control *E. coli* OP50 were subjected to a high temperature of 37 °C. Worms grown on *Chryseobacterium* sp. CHNTR56 MYb120 had increased survival at all time points measured, compared with worms grown on *E. coli* OP50, with a high level of significance (*p* = 0.0605, Mantel–Cox test) (Figure 1b). Next, we sought to find the effect of *Chryseobacterium* sp. CHNTR56 Myb120 on the motility of the worms by counting the number of head thrashes. In contrast to the positive effect on the lifespan of the worms, worms grown on *Chryseobacterium* sp. CHNTR56 MYb120 exhibited a reduced median motility of 56 head thrashes per minute as compared with those grown on *E. coli* OP50, which had a median motility value of 75 head thrashes per minute (Figure 1c). The effects of *Chryseobacterium* sp. CHNTR56 MYb120 on worm health have been studied previously as well, and the tendency of the worms to have high selectivity for such bacteria in their native microbiome community has been highlighted [42]. The trade-off between increased lifespan and reduced motility could be a key factor governing the selectivity of this species by *C. elegans*.

### 3.2. The Influence of Native Member Chryseobacterium sp. CHNTR56 MYb120 on the Metabolome of the Young Adult C. elegans Worm

**Figure 2:**
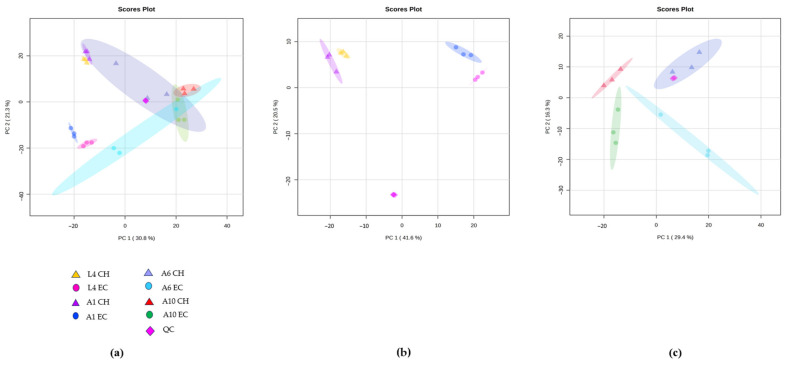
Principal component analysis (PCA) to study the separation of groups based on age and fed bacterium, either *E. coli* OP50 or *Chryseobacterium sp*. CHNTR56 MYb120. (**a**) PCA 2D score plot of the eight groups along with pooled QC samples; (**b**) PCA 2D score plot of the early life-stage groups; (**c**) PCA 2D score plot of the mid–late life-stage groups. (EC: *E. coli* OP50; CH: *Chryseobacterium* sp. CHNTR56 MYb120; L4: L4-young adult stage; A1: adult day 1 stage; A6: adult day 6 stage; A10: adult day 10 stage).

To better-understand the mechanisms underlying the observed phenotypic variations, we performed global metabolomics to capture the metabolic responses of worms grown on *E. coli* OP50 or *Chryseobacterium* sp. CHNTR56 MYb120. We then used principal component analysis (PCA) to assess metabolome differences across the life stages. Based on the PCA results, we found that there was a distinct clustering of the groups based on the type of microbiome species fed to the worms (Figure 2a). The microbiome groups were completely separated along the first principal component axis (PC1), which captured 41.6% of the variation between samples, along with a tight clustering of the pooled QC sample, indicating system reproducibility (Figure 2a).

**Figure 3:**
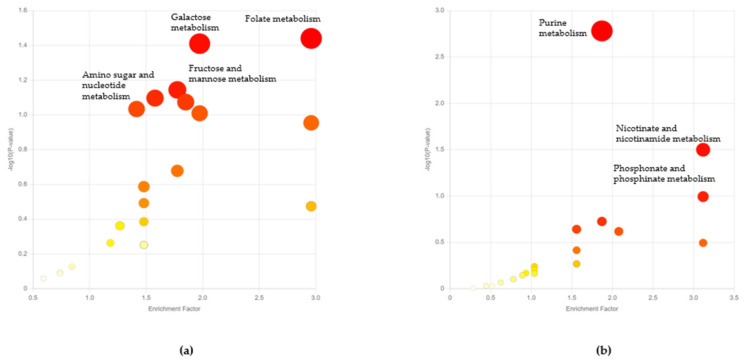
Functional analysis scatter plots for early life-stage worms grown on *Chryseobacterium* sp. CHNTR56 MYb120 versus *Escherichia coli* OP50. (**a**) Functional analysis at the L4-young adult stage of the worm; (**b**) functional analysis at the adult day 1 (A1) stage of the worm.

One key observation that can be visualized from the PCA plot is the clear separation of clusters in the initial life stages of the worm (Figure 2b). Since the influence of the microbiome outweighed that of the life stages, the rest of our analysis pertained to microbiome-based differences at each life stage. We then performed functional analysis to identify the differential pathways at these initial life stages of worms grown on different bacteria [38]. Functional analysis revealed differential folate biosynthesis, galactose, fructose, and mannose metabolisms at the L4-young adult stage, and differential purine, nicotinate, nicotinamide, phosphonate, and glutathione metabolism at the early adult day 1 stage (Figure 3a, Supplementary Table S2a,b). Excessive microbial folate biosynthesis has been linked to reduced lifespan in *C. elegans* grown on *E. coli* [43,44]. Sugar metabolism linked to galactose, fructose, and mannose has been studied in *C. elegans* as well [16]. Galactose is found to be a part of important glycan molecule backbones and is usually metabolized to glucose-1-phosphate [45]. On the other hand, fructose in lower quantities was found to improve the lifespan of *C. elegans* [46]. Upregulated purine metabolism genes were observed in long-living *C. elegans* mutants (i.e., *daf-2* and *eat-2*) [47] and higher levels of purine and nicotinamide intermediates are known to be elevated in early life stages [48].

**Figure 4:**
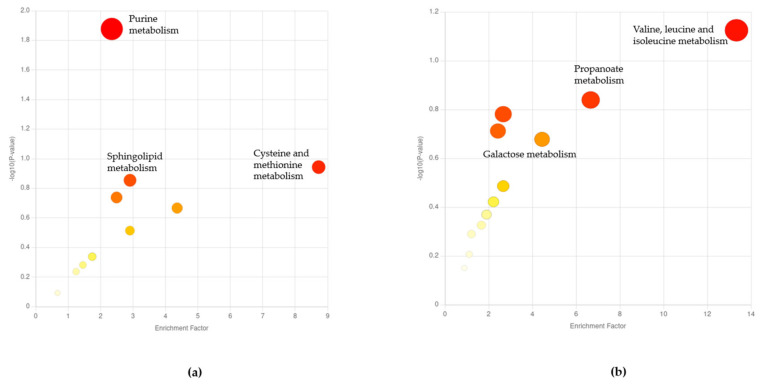
Functional analysis scatter plots at mid- and late life stages. (**a**) Functional analysis at the mid-life, adult day 6 (A6) stage of the worm; (**b**) functional analysis at the late life stage, adult day 10 (A10) stage of the worm.

The change in metabolic response with age was determined by comparing the adult stages on day 6 and day 10. As expected from our PCA results, the effect of the microbiome on the later life stages was not as significant as that on the early life stages, and *Mummichog* results were only obtained at a lower *p*-value of 0.1. Functional analysis revealed that the most significant differential pathways at the adult day 6 stage were purine, cysteine, and methionine metabolisms (Figure 4a). At the adult day 10 stage, there was a change in valine, leucine, and isoleucine metabolisms, and in the propanoate pathway (Figure 4b). This is consistent with other studies, which have found a decline in the levels of these pathways and amino acid levels as *C. elegans* worms age [19,47,48]. In summary, this analysis revealed that the metabolome of *Chryseobacterium* sp. CHNTR56 MYb120-fed worms is distinct to that of the *E. coli* OP50-fed worms, specifically in early life stages. This distinction could explain the differences in healthspan and lifespan, which were observed at an early age.

### 3.3. Chryseobacterium sp. CHNTR56 MYb120 Upregulates the Abundance of Central Carbon Metabolism Intermediates in C. elegans during the Early Life Stages

**Figure 5:**
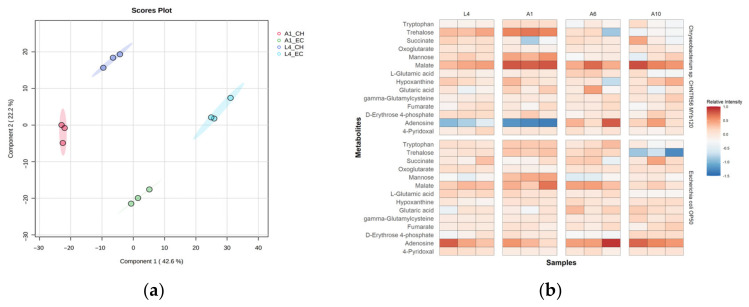
Analysis of key metabolite drivers of longevity affected by the microbiome at different life stages. (**a**). PLS-DA score plot showing separation of microbiome groups along the first component; (**b**) heatmap of top shortlisted metabolites correlated with longevity. (EC: *E. coli* OP50; CH: *Chryseobacterium* sp. CHNTR56 MYb120; L4: L4-young adult stage; A1: adult day 1 stage; A6: adult day 6 stage; A10: adult day 10 stage).

Since our analysis revealed that there is a significant differential response in the metabolism of the worm in the initial stages as compared with the final stages, we further narrowed down our focus to these initial stages of the worm lifecycle. To find the metabolites most affected in the early life stages under the influence of the two different bacteria, PLS-DA was performed (Figure 5a). The top 15 annotated metabolites associated with longevity in *C. elegans* were shortlisted. The set of metabolites upregulated in the early life stages of *Chryseobacterium* sp. CHNTR56 MYb120-fed worms were trehalose and malate, belonging to the central carbon metabolism. 

Among the listed metabolites, trehalose and malate were upregulated in the initial stages of life of the worm metabolism, with the highest levels of abundance being in adult day 1 worms (Figure 5b). Trehalose is a known longevity modulator and provides heat resistance by forming a protective layer on the cells and promoting autophagy [14,29]. A focused study on the effects of added malate and fumarate in *C. elegans* found that malate increased the lifespan and thermotolerance in wild-type worms [49]. This is consistent with our own heat resistance results, which showed that fewer *Chryseobacterium* sp. CHNTR56 MYb120-fed adult day 1 worms died over the span of 10 h as compared with *E. coli* OP50-fed worms. To verify if the heat resistance results also correlated with the life stage of the worm, we performed the heat resistance assay on older worms as well. The results showed that the younger adult worms (day 1) showed better heat-stress resistance compared with older worms (day 3), suggesting a correlation with the levels of trehalose and malate produced at each stage (Supplementary Figure S1). Additionally, it was found that the positive responses conferred by trehalose and malate diminished in the absence of the glyoxylate shunt pathway, as seen in *gei-7* RNAi knockdown worms [18,49]. Altogether, these data suggest that the glyoxylate shunt is active in *Chryseobacterium* sp. CHNTR56 MYb120-fed worms compared with *E. coli* OP50-fed worms.

### 3.4. Isotope Labeling Analysis Reveals the Influence of Chryseobacterium sp. CHNTR56 MYb120 on the Upregulation of Desiccation-Tolerance Pathways in Worms

**Figure 6:**
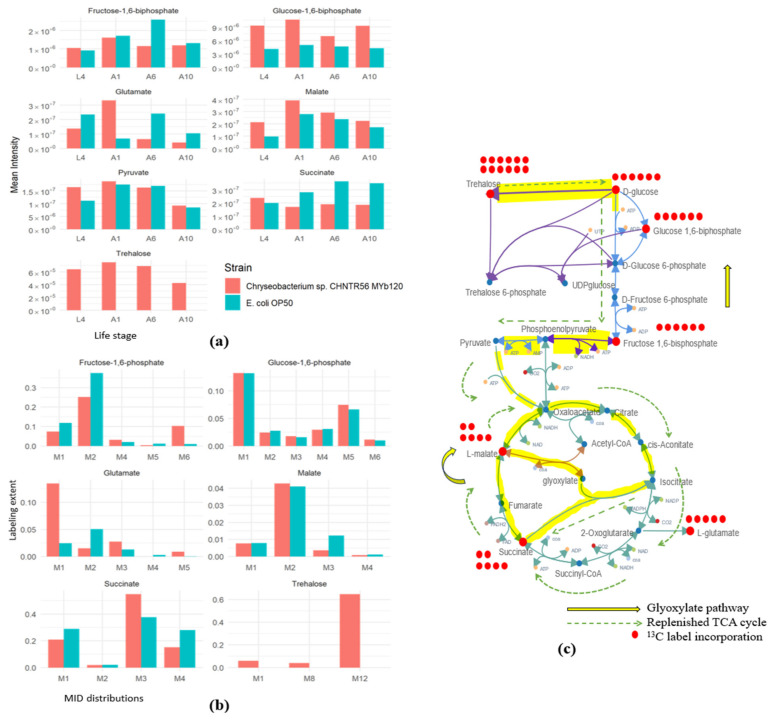
Labeling activity of U-^13^C glucose along the central carbon metabolic pathway intermediates. (**a**) Total labeling extent of central carbon metabolism intermediates for the different life stages of worms grown on *Chryseobacterium* sp. CHNTR56 MYb120 versus *E. coli* OP50; (**b**) MID distributions of key central carbon metabolism intermediates at the L4 stage of the worms; (**c**) diagram of the proposed pathway activities in part of the central carbon metabolism of worms grown on *Chryseobacterium* sp. CHNTR56 MYb120 showing extracted labeling activity. (L4: L4-young adult stage; A1: adult day 1 stage; A6: adult day 6 stage; A10: adult day 10 stage; M_n_—isotopologue distribution, where n is the number of carbon atoms in each metabolite, and n = 1…n).

To better-understand pathway activity at the different life stages and to identify shifts towards the glyoxylate shunt, isotope labeling analysis using U-^13^C-glucose was performed. Labeled versus unlabeled samples were compared in pairs to find those metabolites having a mass shift equivalent to multiples of the atomic mass of the label (i.e., ^13^C = 1.00335 Da) within a narrow window of retention time [37]. Finally, the MID distributions for those isotopologues exhibiting a fold change in intensity greater than 1.5 compared with the unlabeled features were extracted. While comparing the extent of labeling of key intermediates of the central carbon pathways, we observed that trehalose was labeled only in the *Chryseobacterium* sp. CHNTR56 MYb120-fed worms (Figure 6a,b). As mentioned previously, it was discovered that trehalose was produced as a product of the replenishment of glucose facilitated by the glyoxylate pathway in *C. elegans* [18]. Under such conditions, higher levels of malate are produced through a combination of glyoxylate and succinate, the excess of which is converted back to oxaloacetate and is driven towards gluconeogenesis. When looking at the key intermediates of the central carbon pathways, fructose-1,6-diphosphate had higher labeling in the early L4 life stages, while the trehalose precursor glucose-1,6-biphosphate had higher labeling in the adult day 1 stage for *Chryseobacterium sp.* CHNTR56 MYb120-fed worms (Welch’s t-test, *p* < 0.05) (Figure 6a). Furthermore, labeling of the trehalose intermediate was also higher in the adult day 1 stage with a complete absence of labeling in *E. coli* OP50 (Figure 6a). We hypothesized that trehalose production was induced by *Chryseobacterium* sp. CHNTR56 MYb120 in the early stages of the worm’s life along with the activation of the glyoxylate pathway. Since the TCA cycle and glyoxylate pathway share intermediates, following the labeling patterns of these cycles does not yield a very clear picture of their activity [50]. In such cases, glutamate acts as a good proxy for the TCA cycle as it is produced from 2-oxoglutarate, which is solely a part of the TCA cycle (Figure 5c). The labeling pattern of glutamate revealed higher labeling in the adult day 1 stage, suggesting a replenishment of this pathway during this period (Figure 5a). Furthermore, the glutamate levels were lower in the L4 samples, which were sampled at the end of the L4 stage, suggesting glyoxylate activity during the transition between these two stages of the young adult worms (Figure 6a). A previous study showed that dauer *C. elegans* larvae switched to the glyoxylate cycle to generate sugars under conditions of restricted glycolysis activity [18]. Under such conditions, the glyoxylate cycle uses acetyl-coA produced from alternate sources such as acetate or fatty acids to replenish the TCA cycle, which, in turn, facilitates the production of trehalose by feeding into gluconeogenesis [17,18]. Thus, it is suggested that *Chryseobacterium* sp. CHNTR56 MYb120 extends the lifespan of *C. elegans* by upregulating the glyoxylate and trehalose synthesis pathways in the initial life stages (Figure 6c). Both are essential pathways to provide desiccation tolerance to the worms under conditions of stress [18], as seen in our heat-stress resistance assay with and without trehalose supplementation (Figure 1b, Supplementary Figure S2). While the glyoxylate shunt was discovered years ago, its role in the context of host–microbiome interactions has not been demonstrated before. 

## 4. Discussion

The characterization of the native microbiome of *C. elegans* has provided new possibilities for using this worm model to study host–microbiome interactions in addition to its longstanding popularity as a model for aging and diseases [22]. This native microbiome community is dominated by *Proteobacteria*, *Bacteroidetes*, *Actinobacteria*, and *Firmicutes*, which colonize *C. elegans* at different capacities and exhibit striking differences in the life history traits of the worm [42]. *Chryseobacterium* sp. CHNTR56 MYb120 is a member of the *Bacteroidetes* phylum and is a moderate colonizer of wild-type *C. elegans*, conferring a lower developmental rate of growing worms [42]. Previous works found that *Chryseobacterium* sp. CHNTR56 MYb120 was an important community member of the worm’s native microbiome that co-facilitated the production of Vitamin B6 with *Comamonas* sp. 12022 MYb131 and demonstrated the greatest maximum increase in lifespan with *Chryseobacterium* sp. CHNTR56 MYb120 [26]. We demonstrated that the native microbiome member *Chryseobacterium* sp. CHNTR56 MYb120 increased the lifespan of CF512 worms by 18% compared with the *E. coli* OP50 control, with a corresponding increase in trehalose production. It was shown that a concentration of 5 mM trehalose added at the young adult stage increased the lifespan of wild-type worms by 32% [29]. It is not expected that this concentration is achieved in vivo in the worm. Correspondingly, from global metabolomics analysis, worms fed with *Chryseobacterium* sp. CHNTR56 MYb120 showed the highest concentrations of trehalose at the early L4-young adult and adult day 1 stages of the life cycle, depicting that this early spike in trehalose concentrations had a causal effect on overall lifespan extension, which has been described elsewhere [29]. 

Previous reports found that branched-chain amino acids (BCAAs), isoleucine, leucine, and valine are seen to decrease, while phosphocholine, glycerophosphocholine, and glycine are all seen to increase in relative concentrations in wild-type worms as worms develop into the adult stage [11]. However, the influence of the microbiome on these developmental stages has not been profiled. Our current work revealed the metabolic signatures are influenced by a longevity-promoting native microbiome member. 

Trehalose is a naturally occurring disaccharide consisting of two molecules of glucose. It is produced by several organisms ranging from bacteria to invertebrates; however, mammals lack the genes required to synthesize trehalose [51]. In *C. elegans*, trehalose is synthesized via the action of three genes, *tps-1*, *tps-2*, and *gob-1* [52], and is known to confer stress resistance and longevity [14,29]. A study showed that increased internal trehalose levels either through dietary supplementation or via the mutation of trehalase enzyme genes protected against high glucose toxicity and improved the lifespan of wild-type worms in a DAF-16-dependent manner [53]. On the supplementation of trehalose to wild-type L1 worms, it was found that trehalose synthesis is still in play, and this additional synthesis is required to convey the full effect of added trehalose supplementation on lifespan and stress resistance [14]. Similarly, we suggest that *Chryseobacterium* sp. CHNTR56 MYb120 induced increased synthesis of trehalose production in *C. elegans*. An analysis of the transcriptomic response of L4-young adult stage N2 worms fed on *Chryseobacterium* sp. *CHNTR56* MYb120 vs. *E. coli* OP50 showed the upregulation of trehalose phosphate synthase enzymes *tps-1* (FC = 1.87; *p* = 0.01) and *tps-2* (FC = 1.41; *p* = 0.09) [25,52]. Furthermore, trehalose is strongly associated with increased heat-stress resistance in *C. elegans* [14,18]. This was clearly shown in our heat resistance results, where worms grown on *E. coli* OP50 exhibited decreased survival earlier in their life (adult day 1 versus day 3). Apart from this, it was discovered that trehalose upregulates autophagy-related genes in a *daf-16-*dependent manner [14]. Interestingly, autophagy was found to increase the lifespan of *C. elegans* via the reduction of protein aggregation [54], and was discovered as a mode of lifespan extension in trehalose-fed worms [10,29].

In summary, we characterized phenotypes associated with the growth of worms on a native microbiome member, *Chryseobacterium* sp. CHNTR56 MYb120, and then employed global metabolomics to associate changes in metabolites throughout the lifecycle of the worm with the observed phenotypes. We identified trehalose as a metabolite of interest, as illustrated in the literature, which was differentially upregulated in the *Chryseobacterium* sp. CHNTR56 MYb120-fed worms, especially early in life. We further employed ^13^C isotope labeling analysis to pinpoint the pathway activity leading to the production of trehalose. Therefore, we can confirm with more certainty that *Chryseobacterium* sp. CHNTR56 MYb120 upregulated the production of trehalose in the early stages of the worm lifecycle by enabling a shift in the central metabolism from the TCA cycle to glyoxylate metabolism. Trehalose has been historically demonstrated to increase the lifespan of wild-type worms, along with improved stress resistance [14,29,53]. However, this is the first report that depicts the influence of the microbiome on the production of this important metabolite. The use of trehalose as a supplement to dietary sugar, especially for people with diabetes, has recently been explored [55]. The role of the microbiome in regulating trehalose metabolism increases our insights into the range of beneficial effects extended by the gut microbiome, which could have a direct effect on the healthspan and lifespan of the host. 

## 5. Conclusions

Our work demonstrates microbiome-enriched trehalose production in *C. elegans* worms. Worms grown with *Chryseobacterium* sp. CHNTR56 MYb120 exhibited increased trehalose levels in the early adult stages with a corresponding decrease in glucose abundance levels (Figure 5b and Figure 6a). To further study the changes in pathway activity based on these findings, global isotope labeling was employed [40]. Isotope labeling with U-^13^C glucose showed decreased labeling of glycolysis pathway intermediates along with a corresponding increase in labeling of glyoxylate pathway intermediates in the early life stages of the worm, suggesting a metabolic shift which leads to the downstream production of trehalose. Finally, studies using labeled acetate could help further outline glyoxylate pathway activity in correlation with trehalose production in our *C. elegans* microbiome aging model. Finally, future global metabolomics studies that include other native microbiome members could help contribute to our understanding of interbacterial interactions in the context of the host.

## Data Availability

The raw data for the MS1 and MS/MS analyses can be downloaded from our server from the following link: http://gofile.me/4esAc/YG1mBDauB (accessed on 11 August 2023).

## Supplementary Materials

The following supporting information can be downloaded at: https://www.mdpi.com/article/10.3390/metabo13080953/s1, Table S1: LC-MS/MS gradient list; Table S2: Mummichog pathway enrichment table; Table S3: DDA annotation results for MS/MS data; Supplementary Figure S1: Heat stress assay for adult day 3 worms; Supplementary Figure S2: Heat stress assay for adult day 1 worms with and without trehalose supplementation.
