# Supplementary figures and images for: The Native Microbiome Member Chryseobacterium sp. CHNTR56 MYb120 Induces Trehalose Production via a Shift in Central Carbon Metabolism during Early Life in C. elegans
Source: Metabolites. 2023 Aug 18;13(8):953. doi: 10.3390/metabo13080953 (PMC10456584; doi:10.3390/metabo13080953)

Adult Day 3 *C. elegans* Survival at 37°C

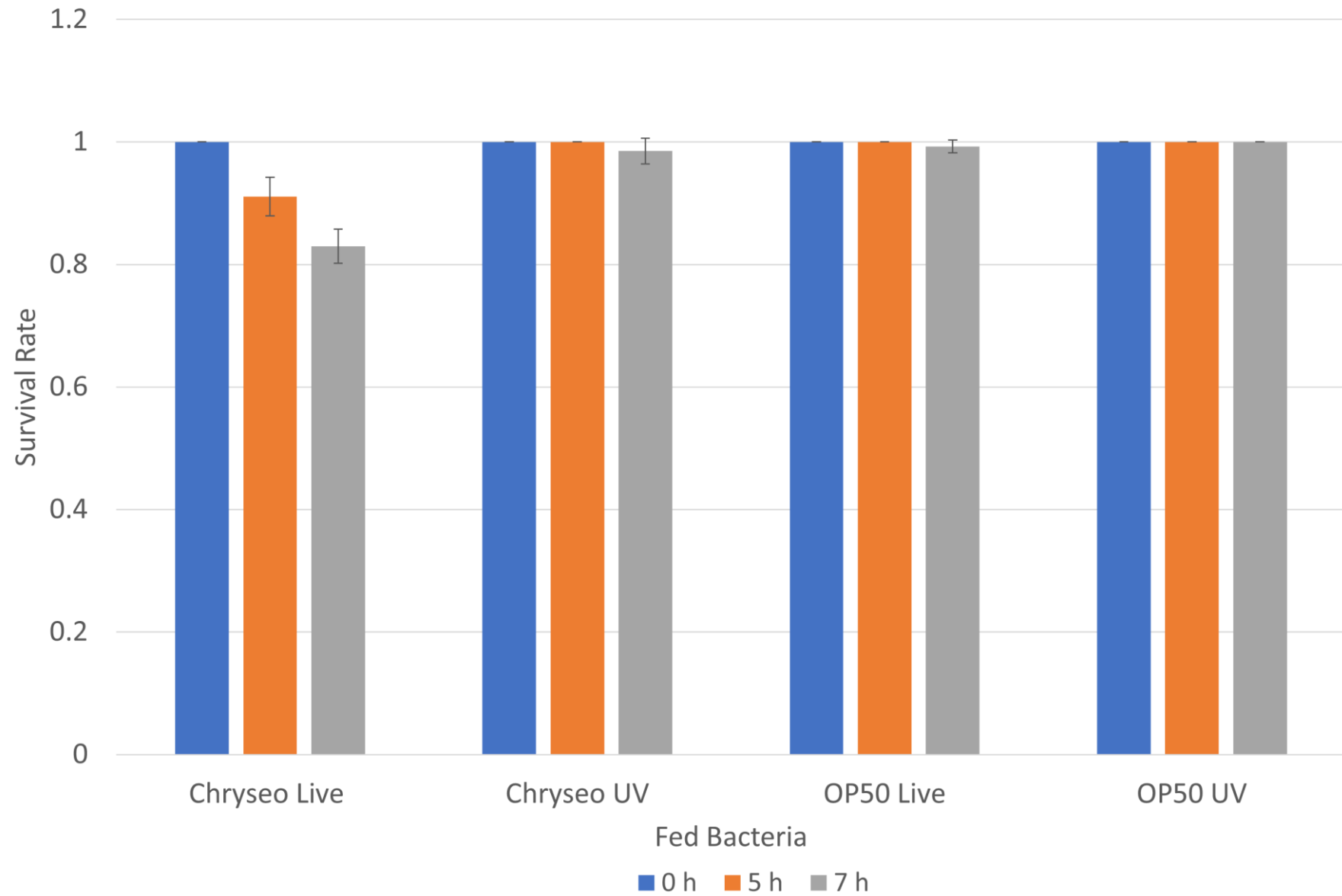

Supplement: Supplementary file 1 [file metabolites-13-00953-s001.zip › Supplementary figure S1.pdf]

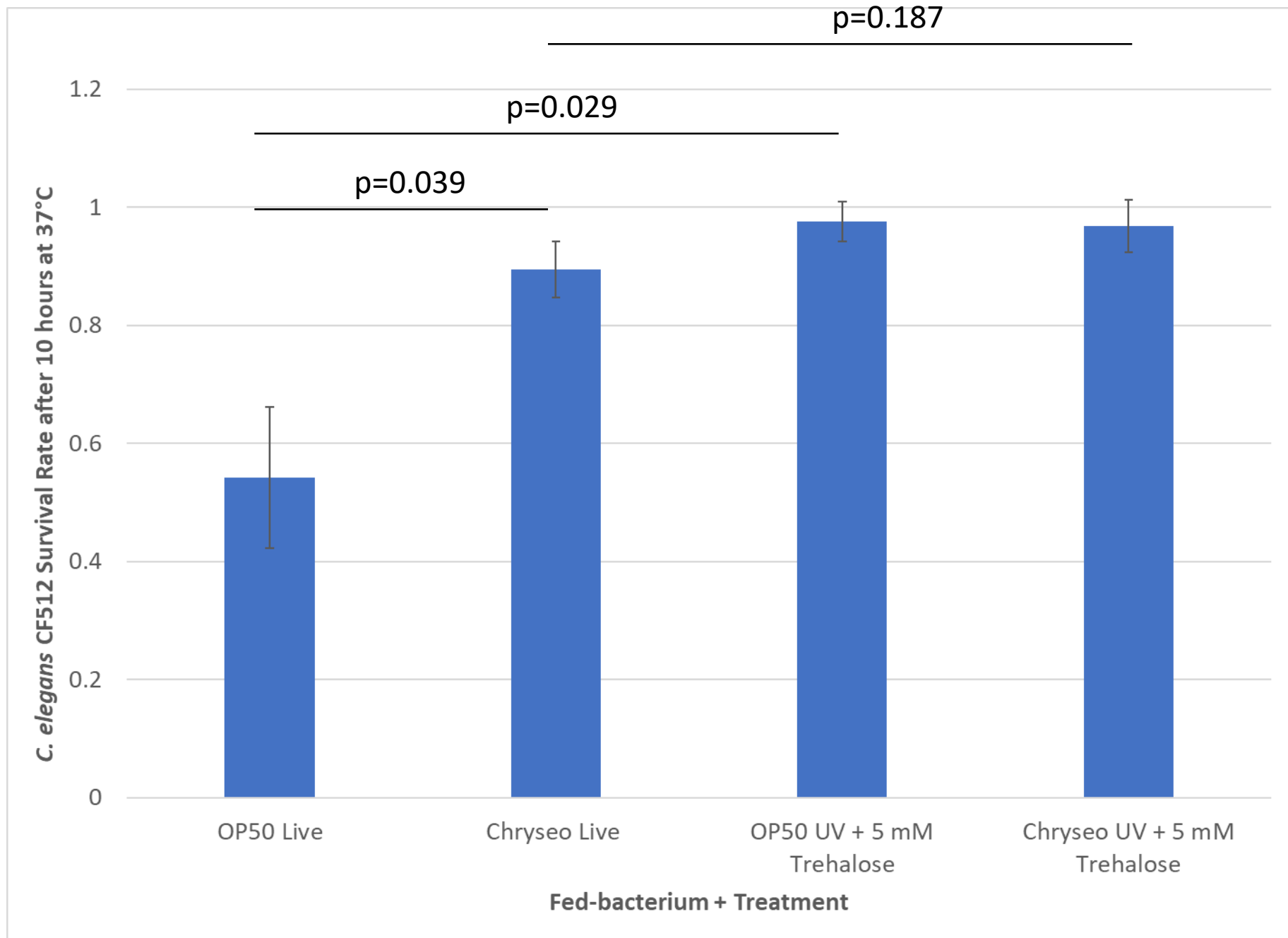

Supplement: Supplementary file 1 [file metabolites-13-00953-s001.zip › Supplementary figure S2.pdf]
